# A short review of application of near-infrared spectroscopy (NIRS) for the assessment of microvascular post-occlusive reactive hyperaemia (PORH) in skeletal muscle

**DOI:** 10.3389/fphys.2024.1480720

**Published:** 2024-11-27

**Authors:** Elizabeth Hendrick, Alexandra Jamieson, Scott T. Chiesa, Alun D. Hughes, Siana Jones

**Affiliations:** Institute of Cardiovascular Science, University College London, London, United Kingdom

**Keywords:** NIRS (near-infrared spectroscopy), post-occlusive reactive hyperaemia, microvascular, skeletal muscle, methods

## Abstract

Near-infrared spectroscopy (NIRS) is an optical technique that can be used to non-invasively interrogate haemodynamic changes within skeletal muscle. It can be combined with a short (3–5 min) arterial cuff-occlusion to quantify post-occlusive reactive hyperaemia (PORH). This technique has utility in tracking changes in vascular health in relation to exercise, disease progression or treatment efficacy. However, methods for assessing PORH vary widely and there is little consensus on methodological approaches such as sampling frequency, correction for adipose tissue or the analysis endpoints. The purpose of this review was to: (1) summarise recent advances; (2) compare different methodological approaches and (3) identify current knowledge gaps and future objectives for use of NIRS for vascular assessment. We propose key areas for future work, including optimising occlusion duration and comparing methods of correction for the ischemic stimulus, standardising methods for adjustment of adipose tissue thickness, cross-device comparisons and establishing a standard for minimum sampling rate. Comparisons with alternative methods of capturing PORH or upstream vasodilatory responses would be valuable. Addressing these methodological considerations will aid our understanding of this useful, non-invasive tool for characterising PORH within skeletal muscle and facilitate interpretation of results across studies.

## 1 Introduction

Post-occlusive reactive hyperaemia (PORH) refers to the increase in blood flow following brief ischemia induced by an arterial occlusion and is an indicator of vasodilatory capacity. Blunted PORH indicates vascular dysfunction and is a predictor of cardiovascular morbidity and mortality (Ishibashi et al., 2006; Paine et al., 2016; Anderson et al., 2011). PORH also provides the stimulus for flow-mediated dilation (FMD) in conduit arteries (Corretti et al., 2002) which also predicts future cardiovascular outcomes (Inaba et al., 2010).

Near-infrared spectroscopy (NIRS) is a non-invasive optical method that measures oxygenated and deoxygenated haemoglobin and myoglobin (referred to as oxy [heme] and deoxy [heme] here, in line with a recent call for standardization but also widely referred to as oxy [Hb + Mb] or deoxy [Hb + Mb]) in small blood vessels and myocytes (Scholkmann et al., 2014). In skeletal muscle, NIRS has been used to detect differences in PORH in peripheral arterial disease (Jarm et al., 2003; Kooijman et al., 1997; Kragelj et al., 2001), coronary heart disease (Monthé-Sagan et al., 2016; Gayda et al., 2015), metabolic syndrome (Gayda et al., 2015), sepsis (Creteur et al., 2007; Mayeur et al., 2011), acute respiratory disorders (Orbegozo Cortés et al., 2016), in smokers versus non-smokers (Siafaka et al., 2007; Zamparini et al., 2015), and between different age groups (Rosenberry et al., 2018; de Oliveira et al., 2020). However, NIRS is relatively under-exploited compared to other tools, such as venous occlusion plethysmography (VOP) or peripheral arterial tonometry (PAT), and methods can vary considerably between studies.

This narrative review aims to describe the application of NIRS for assessment of PORH in skeletal muscle and summarise methods from publications spanning the last 25 years. Publications were identified by screening online databases, MEDLINE and PubMed Central, using search terms ‘post-occlusive reactive hyperaemia’ and ‘near-infrared spectroscopy’ and ‘muscle’. Additional publications were identified through reference lists within the screened publications. We did not aim to undertake a systematic review of the literature or provide an exhaustive list of studies applying NIRS to capture PORH in this mini review. Studies were used as examples to highlight key points discussed. To demonstrate the range of different methods applied, we extracted information about study design, the type of NIRS device used, the signal processing methods and analysis endpoints from the example studies. We did not formally assess the quality of the studies included here. We compared NIRS with alternative methods for assessing PORH and aimed to identify targets for the advancement of NIRS as a vascular assessment tool. A comprehensive comparison of the different methods for capturing PORH is available elsewhere (Rosenberry and Nelson, 2020). A general review of NIRS application to skeletal muscle (Barstow, 2019a) and technical reviews of NIRS technology have also been published previously (Scholkmann et al., 2014).

## 2 PORH assessment using NIRS, data processing and analysis endpoints

NIRS devices are typically applied to the distal portion of a limb to measure PORH. The mechanism of PORH is fairly well understood (Rosenberry and Nelson, 2020). When the pressure inside the small arteries declines, wall circumferential stress drops, withdrawing myogenic tone and causing vasodilation (the Bayliss effect) (Bayliss, 1902). During ischemia, various factors also contribute to vasodilation, including adenosine (Cheng et al., 2000), H^+^ ions (Granger and Shepherd, 1973), prostaglandins (Engelke et al., 1996), K^+^ ions (Shimoda and Polak, 2011), bradykinin (Hornig et al., 1997), endothelium derived hyperpolarisation factor and nitric oxide (NO) (Shu et al., 2015), although the role of NO is debated (Wong et al., 2003). Hyperaemia occurs immediately following cuff release and an increase in shear stress in conduit vessels further promotes re-perfusion via FMD Corretti et al., 2002.

**FIGURE 1:**
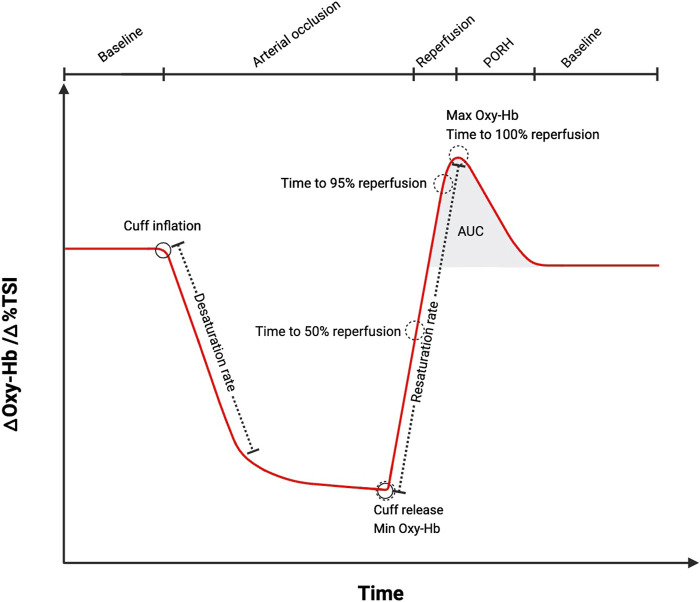
A schematic diagram illustrating change in oxygenated haemoglobin (oxy-Hb, oxy [heme]) or tissue saturation index (TSI, StO_2_) during ischemia and post-occlusive reactive hyperaemia (PORH) measured using near-infrared spectroscopy (NIRS). The most common endpoints that can be extracted from the NIRS signal are labelled. AUC (area under the curve). Schematic created using BioRender.com software, based on measurements performed by the authors and schematics previously presented (Rosenberry and Nelson, 2020).

Haemodynamic changes are observed in NIRS signals as rapid increases in oxy [heme], total [heme] (oxy [heme]+ deoxy [heme]) or tissue saturation/oxygenation index (referred to here as StO_2_ but also commonly referred to as TSI or TOI%). NIRS analysis endpoints can be derived that characterize the changes in these signals, most commonly, they include: reperfusion (resaturation) rate (Kooijman et al., 1997; Creteur et al., 2007; Mayeur et al., 2011; Rosenberry et al., 2018; Cheatle et al., 1991; Cheng et al., 2004; Hammer et al., 1985; Lacroix et al., 2012; McLay et al., 2016a; McLay et al., 2016b), peak response as an absolute tissue saturation value (maximum StO_2_) or peak value of oxy [heme], the magnitude of change (Δ from baseline value to peak value of StO_2_ or oxy [heme]) or a percentage change (Kragelj et al., 2001; Creteur et al., 2007; Rosenberry et al., 2018; Cheng et al., 2004; Hammer et al., 1985; McLay et al., 2016a; McLay et al., 2016b; Chung et al., 2018), time to peak reperfusion following cuff-release or a given percentage of that time (50%, 95% of peak) and the area under the reperfusion curve (AUC reperfusion) (Jones et al., 2022) (Figure 1; Table 1). Desaturation rate during the occlusion or magnitude of desaturation (Δ StO_2_ between rest and minimum StO_2_ at the end of the occlusion) are frequently reported and response beyond the peak PORH (return of signals to resting values/baseline) has been described in some studies (Table 1). The pattern of the reperfusion response and recovery beyond peak have also been quantified (Bopp et al., 2014; Bopp et al., 2011). Patterns of reperfusion and recovery have been modelled using various functions (Bopp et al., 2014; Bopp et al., 2011). In a study where three functions (Gompertz, logistic and exponential) were fit to the same PORH data across 20 healthy subjects, the authors found that the Gompertz function provided the best fit to the oxy [heme] reperfusion data (Bopp et al., 2011). Studies do not typically present all derivable endpoints and terminology for endpoints is variable. Consensus on a set of standard endpoints and on the nomenclature would be beneficial. A limitation is the assumption that the majority of the change in signal is due to changes in blood flow; it is possible that the changes observed are influenced by alterations in oxygen consumption rate in the tissue. Using the total [heme] or StO_2_ signals addresses this limitation to some degree as the changes in deoxy [heme] are incorporated into the signal.

**TABLE 1:** An overview of the methodological aspects of key studies from the past ∼30 years including a list of the endpoints derived from each as well as the signals analysed to retrieve endpoints. A NIRS position indicated as ‘calf’ refers to either the lateral or medial gastrocnemius. A NIRS position indicated as ‘forearm’ refers to either the flexor digitorum profundus or brachioradialis, flexor carpi radialis, flexor. Signals indicates the signals used for the processing (not a report of all the signals that are possible to derive from the specified device used) and nomenclature is as reported in the publication. AUC (area under curve), ATT (adipose tissue thickness), CW (continuous wave), deoxy [heme] (deoxygenated haemoglobin/myoglobin concentration), FD (frequency domain), NS (not specified), oxy [heme] (oxygenated haemoglobin/myoglobin concentration), SD (source-detector), SRS (spatially resolved spectroscopy), tot [heme] index (THI).

	Study design		Device specifics				Signal processing	
First author and year	NIRS position	Occlusion duration (mins)	Source-detector distance (cm)	NIRS type	SD pairs	Sample f (Hz)	Signals	Correct for O_2_ deficit?
Cheatle et al. (1991)	Calf	3–5	3.0–5.0	CW	6	0.05–0.5	oxy [heme]	No
Kooijman et al. (1997)	Calf	5	3.5	CW	NS	NS	oxy [heme]	No
Jarm et al. (2003)	Foot	5	3.5–4.0	CW	1	NS	oxy [heme]	Yes
Kragelj et al. (2001)	Foot	5	4.0	CW	1	1	oxy [heme]	Yes
Cheng et al. (2004)	Calf	≤5	≤2.2	CW	4	NS	StO_2_	No
Creteur et al. (2007)	Hand	3	2.5	CW	1	0.3	StO_2_	Yes
Siafaka et al. (2007)	Hand	3	NS	CW	1	0.3	StO_2_	No
Bopp et al. (2011)	Forearm	5	2.0–3.5	FD	2	NS	oxy [heme] deoxy [heme] tot [heme]	Yes
Mayeur et al. (2011)	Hand	3	1.5	CW	1	0.5	StO_2_	Yes
Lacroix et al. (2012)	Forearm	5	4.5	CW	1	10	oxy [heme] deoxy [heme] tot [heme]	No
Bopp et al. (2014)	Forearm (x3)	5	NS	FD	NS	NS	tot [heme]	No
Fellahi et al. (2014)	Calf & Foot	SpO_2_ 40%	NS	CW	4	0.25	StO_2_ (rSO_2_)	No
Gayda et al. (2015)	Forearm	5	4.5	CW	NS	10	oxy [heme], deoxy [heme] tot [heme]	No
Zamparini et al. (2015)	Calf	SpO_2_ 40%	NS	CW	4	0.25	StO_2_	No
McLay et al. (2016b)	Tibialis anterior	5	2.0 2.5, 3.0 & 3.5	FD	4	2	StO_2_ *(oxy[heme]/tot[heme])*	No
Monthé-Sagan et al. (2016)	Forearm	SpO_2_ 40%	NS	CW	4	0.71	StO_2_ (rSO_2_)	No
Orbegozo Cortés et al. (2016)	Forearm	3	NS	CW	NS	NS	StO_2_ THI	No
Willingham et al. (2016)	Calf and foot	5	Adjusted to ATT (2.5–5.5)	CW	2	10	oxy [heme]	No
Rosenberry et al. (2018)	Forearm	5	NS	FD	NS	NS	StO_2_	Yes
Hammer et al. (1985)	Forearm	5	2.0, 2.5, 3.0 & 3.5	FD	4	25	oxy [heme] deoxy [heme] tot [heme] diff [heme] StO_2_	No
Townsend et al. (2019)	Forearm	5	NS	FD	NS	NS	StO_2_	Yes
de Oliveira et al. (2020)	Forearm	5	3.5	CW SRS	3	10	StO_2_	Yes
Whyte et al. (2022)	Thigh	3	1.25, 2.5	CW	NS	2	StO_2_(Moxy)	No
Gonzalez (2022)	Forearm	5	NS	CW	2	10	StO_2_	Yes
Didier et al. (2022)	Calf	5	2.2, 2.5, 3.0, 3.5	FD	2	50	oxy [heme] deoxy [heme] tot [heme] StO_2_	Yes
Jones et al. (2022)	Calf	3	NS	CW	NS	10	StO_2_	Yes
Desanlis et al. (2022)	Forearm	7	3.0, 3.5, 4.0	CW	2	10	StO_2_ oxy [heme] deoxy [heme] tot [heme]	Yes
Anderson and Park (2023)	calf	5	NS	CW	NS	NS	StO_2_	Yes
McGranahan et al. (2023)	calf	5	3.5	CW	2	10	oxy [heme] StO2%††	No
Dellinger et al. (2023)	Thigh	5	3.0, 3.5, 4.0	CW SRS	3	10	StO_2_	No
Theurot et al. (2023)	Forearm	5	NS	NS	NS	10	oxy [heme] deoxy [heme]	No
✓					✓		✓			
✓	✓†	✓				✓	✓			
✓	✓						✓			
	✓	✓			✓					
✓	✓	✓					✓	✓		
✓	✓					✓	✓			
	✓	✓		✓	✓					
✓										
	✓	✓		✓						
✓	✓									
✓	✓						✓	✓		
✓	✓	✓		✓					✓	
✓				✓		✓		✓		
✓							✓			

^a^ratio of AUCs, to give response adjusted for desaturation.

^b^hyperaemic reserve (baseline-max StO2).

^††^StO2 oxygenation percentage derived through calibration to the range of oxy [heme].

When conditions where there is vascular dysfunction, such as PAD, are compared to healthy participants, NIRS endpoints tend to be impaired (e.g., slower reperfusion rates, prolonged recovery times and smaller magnitude of change (Δ minimum to maximum during PORH)) (Kooijman et al., 1997; Kragelj et al., 2001; Creteur et al., 2007; Cheatle et al., 1991). It is unlikely that specific physiological mechanisms underpinning PORH (described above) can be inferred from specific analytical endpoints. However, combining NIRS with additional measurement methods in the future may permit these different aspects to be delineated to some extent.

## 3 Study design

### 3.1 NIRS measurement site

Theoretically, any portion of a limb or tissue where an arterial occlusion can be applied safely can be a measurement site. Typical measurement sites are: the forearm (brachioradialis or flexor digitorum profundus), calf (gastrocnemius or tibilias), hand (thenar eminence) and foot (Table 1). Consideration of the research question and participant population is important prior to muscle group selection. The thenar eminence has been recommended due to low levels of overlying subcutaneous adipose tissue thickness (ATT), minimal oedema, and good reproducibility in healthy subjects (Mayeur et al., 2011; Gómez et al., 2009). If an exercise intervention is being tested, a muscle group which is suitable for training should be chosen. Similarly, if the participant population is likely to have excess adiposity, a site with less ATT is preferable.

Different anatomical sites may differ in terms of metabolic and vascular properties, muscle thickness, fibre type proportion and overlying ATT. These differences make comparisons between studies challenging. PORH has been compared across different measurement sites in the same individuals by Gomez et al. (Gómez et al., 2009). They found that baseline oxy [heme] and the magnitude of oxy [heme] desaturation and oxy [heme] re-saturation were lower in the forearm compared to the hand; however, we do not know the extent to which these reflect differences in microvascular behaviour or differences in overlying tissue properties that impact NIRS measurements. Future work on these questions would be useful. Use of multi-channel devices at different points along the same muscle during PORH could permit intra-muscle similarities/differences to be examined. Consideration of distance between measurement site and the cuff occlusion site may also be important, but we are unaware of any published work that has explored this.

Participants who undertake PORH measurements often find the occlusions uncomfortable, and at times, painful. Lacroix et al. reported that the forearm occlusion was less uncomfortable than lower limb occlusion (Lacroix et al., 2012) but there has been limited work exploring the effect of discomfort on results; if investigators frequently find it necessary to exclude participants who are unable to complete the pre-specified occlusion duration, this may introduce selection bias into presented results. Future work exploring participant discomfort during arterial occlusions, perhaps via incorporation of pain scale questionnaires, could provide valuable practical insights.

### 3.2 Measuring adipose tissue thickness and adjusting source-detector distance

If ATT is greater than half the distance between the NIRS source and detector, little or no signal from skeletal muscle will be detected (Van Beekvelt M. et al., 2001). Most NIRS devices have fixed distances of 2–4 cm, but some have multiple light sources or detectors fixed in series (multi-distance devices). Using longer source-detector distances allows greater sampling depth but at the cost of reduced signal.

Despite being a well-recognised limitation, ATT is not routinely measured or corrected for in studies that use NIRS. Measurement of subcutaneous ATT overlying the muscle of interest can be done using callipers or an appropriate ultrasound device. Out of the studies in Table 1, three included a correction method for ATT, one adjusted source-detector distance to at least twice that of an individual’s ATT (41), and two used statistical adjustment to account for ATT (36, 37). In a study where total [heme] (frequency domain NIRS) and ATT were measured across different muscles, results suggested ATT correction equations for oxy [heme] and deoxy [heme] may be specific to the muscle of interest (Craig et al., 2017). In work where the reperfusion response has been modelled using sigmoidal or exponential functions, Bopp et al. found that the calculated time constants representing the reperfusion curve (τ1) or the return to baseline curve (τ2) were far less affected by ATT than the parameters reflecting the magnitude of change (Bopp et al., 2014; Bopp et al., 2011). This suggests that modelling the kinetics of the PORH may be a useful way to circumvent the impact of ATT on the analysis endpoints. Further studies examining the effect of ATT on quantification of PORH would be useful.

### 3.3 Occlusion duration

The magnitude of the ischemic stimulus is an important determinant of PORH. The most common occlusion durations reported are three or 5 min. A limitation of using pre-set occlusion durations is that rates of deoxygenation may vary considerably across individuals. In a recent model, the StO_2_ deficit was directly associated with the reoxygenation rate; as oxygen deficit increased, reoxygenation rate increased (Anderson and Park, 2023). McLay et al. (McLay et al., 2016a) found that in all of their participants, a desaturation plateau was reached before 5-min of occlusion, but 3-min occlusions in other studies have resulted in varying desaturation levels (Mayeur et al., 2011). A major advantage of NIRS, compared to other techniques, is that changes in tissue saturation can be recorded during the occlusion and a rate of desaturation (oxygen consumption rate) can be calculated. This allows users to standardize the ischemic stimulus or account for it in a post-processing step. Rosenberry et al. (Rosenberry et al., 2018) highlighted the potential importance of differences in desaturation. When they adjusted the occlusion duration in young and old individuals to standardize the level of desaturation, they found that age-related differences in PORH were attenuated. Some studies present the amplitude of oxy [heme] change as a percentage of the occlusion stimulus to circumvent this problem (Jarm et al., 2003; Kragelj et al., 2001; McGranahan et al., 2023). Adjusting occlusion duration so that a pre-specified reduction in oxy [heme] or StO_2_ is achieved is an alternative and several studies have employed this approach (Monthé-Sagan et al., 2016; Mayeur et al., 2011; Zamparini et al., 2015; Fellahi et al., 2014). Taking baseline StO_2_ value into account may also be beneficial, since this can differ between participants due to varying basal metabolic rates and vascular architecture at the measurement site.

## 4 Technical considerations

### 4.1 NIRS devices and sampling frequency

Broadly speaking, a NIRS device works by emitting a known intensity of NIR-light (650–950 nm) at two or more wavelengths into tissue, measuring the intensity of returning light at each wavelength. Based on differential absorption patterns of each wavelength by oxy [heme] and deoxy [heme], the device estimates the concentration (or concentration change) of each chromophore (Jones et al., 2022). This simple technology has been developed into several different device types: continuous wave (CW), frequency-domain (FD) and time domain (TD) devices, reviewed elsewhere (Barstow, 2019b). Thus far, only FD and CW devices appear to have been employed for PORH measurements (Table 1). CW devices are simple and cost-effective but are limited in only providing information about relative chromophore concentrations. During PORH, as we are interested in the response to ischemia, this is not a major limitation. FD devices can account for light scattering and therefore measure absolute concentrations. Direct comparisons between different types of NIRS devices during PORH would be valuable.

Multi-distance CW-NIRS devices can use spatially resolved spectroscopy (SRS) to calculate a StO_2_ (Artinis Medical Systems TN, 2022). SRS addresses some of the problems relating to variations in ATT, and StO_2_ is frequently used for PORH. There are some variations in the design of devices applying SRS. Some devices use multiple light sources and a single detector while others use a single light source with multiple detectors to quantify linear change of light attenuation against distance (Suzuki et al., 1999). We have not found studies comparing different light source-detector arrangements for PORH. A source of confusion is that the term ‘StO_2_’ is used synonymously with ‘TSI’ (tissue saturation index), ‘TOI’ (tissue oxygen index) and ‘SmO_2_’ (muscle tissue oxygen saturation) in the literature. Also, some authors calculate StO_2_ as oxy-[heme]/(oxy [heme] +deoxy [heme])*100% in the absence of SRS. Consensus on use of these terms has previously been called for and would be beneficial.

NIRS devices typically have high temporal resolution (>1 Hz), although sampling rates as low as 0.25 Hz have been applied successfully (Mayeur et al., 2011; Zamparini et al., 2015; Cheatle et al., 1991; Fellahi et al., 2014). In contrast, VOP is limited to one measurement every 5–10 s. Higher sampling frequency is beneficial for capturing changes that occur immediately on cuff release and allows identification of pulsatile changes in the oxy-Hb signal at rest. Reciprocal changes in oxy [heme] and deoxy [heme] signals and absence of pulsatility are useful criteria to indicate successful arterial occlusion.

### 4.2 Myoglobin contribution to the NIRS signals during PORH

The absorption spectra of oxy- and deoxy-myoglobin (Mb) within myocytes and circulating oxy- and deoxy [heme], are very similar. It has been estimated that, in contracting muscles, the Mb contribution to the signal ranges from 20% to 80% (Lai et al., 2009; Davis and Barstow, 2013). The possibility that the oxy [heme] contribution is not constant, changing with varying degrees of tissue hypoxia, has implications for PORH. This was recently explored through combining an experimental canine model with a computational model of muscle O_2_ transport and utilization (Koirala et al., 2023). This analysis indicated that Mb contributed ∼20–30% of NIRS signals at rest, but when O_2_ delivery was reduced, the proportion of deoxy-[heme] signal due to Mb increased to 50% and the contribution to the oxy [heme] signal increased from 0% to 30%. This suggests analysis endpoints derived using only the oxy [heme] NIRS signal would be influenced by changing Mb contributions throughout desaturation or resaturation. Considering changes in the total [heme] signal or a StO_2_ signal would address this problem to some extent as it is unlikely that the total concentration of myocyte Mb changes during the ischemia-reperfusion manoeuvre.

Another consideration is that type I (slow twitch) muscle fibres typically contain higher concentrations of Mb than type II (fast twitch) muscle fibres (Bekedam et al., 2009). Variation exists in the typical fibre-type proportion between muscles; for example, the soleus muscle is known to be comprised of mostly type I fibres while triceps contain mostly type II fibres (Talbot and Maves, 2016). This could be important when comparisons are made across different muscle groups. However, it is also known that muscle fibre type can be altered with training, which may pose difficulties in comparing untrained with trained populations or pre-versus post-exercise intervention (Talbot and Maves, 2016). Recent work in elite cyclists suggests that they may overall have reduced Mb concentration compared to active controls (Jacobs et al., 2023). Furthermore, it has been shown that Mb concentration is greater in older compared with young adults (Möller and Sylven, 1981); this has implications for comparisons of PORH by age.

### 4.3 Reproducibility of PORH parameters measured by NIRS

Several studies have reported the reproducibility of NIRS PORH(13, 32, 34, 41, 44, 56). Test-re-test reproducibility for temporal, absolute range and reperfusion recovery slope parameters ranges from adequate to excellent (intra-class correlation coefficients (ICC) between 0.5 and 0.9) in the leg (McLay et al., 2016b; Willingham et al., 2016; McGranahan et al., 2023) and adequate to good in the arm (ICC 0.6–0.84) (Lacroix et al., 2012). Time to peak hyperaemia was less reproducible than time to 95% peak, since the duration of the last 5% of reperfusion can vary substantially (Willingham et al., 2016). Mclay et al. directly compared repeatability of PORH by NIRS and FMD in the leg and found better intra- and inter-day reproducibility for the NIRS measurements (McLay et al., 2016b). These studies used 5-min occlusion durations and did not adjust for oxygen deficits (desaturation) during the occlusion. Mayeur et al. (Mayeur et al., 2011) reported better reproducibility when using a 40% desaturation target versus a set 3-min duration in the forearm (coefficient of variation 5.9% versus 10.5%). Further studies exploring reproducibility of NIRS-measured PORH following different occlusion durations will be valuable in establishing the precision of the technique.

As NIRS technology develops, it will be important to assess repeatability of newer devices for assessment of PORH. For example, use of StO_2_% measured by SRS in place of raw oxy [heme] and deoxy [heme] data for PORH is becoming more widespread and it will be important to establish the reproducibility of StO_2_% in quantifying PORH. Assessing the precision of more advanced modelling techniques for quantifying PORH, such as methods described by Bopp et al. (Bopp et al., 2014), will also be important.

## 5 NIRS agreement with alternative methods

VOP is widely used to assess PORH (Rosenberry and Nelson, 2020; Wilkinson and Webb, 2001). Studies comparing NIRS with VOP report mixed results. (De Blasi et al., 1994) reported excellent correlation between NIRS and VOP (r = 0.93) for forearm blood flow at rest and after 6-min ischemia. Others have also found that resting measures of blood flow using the venous occlusion method by NIRS and strain-gauge plethysmography are strongly correlated (Edwards et al., 1993; Van Beekvelt MC. et al., 2001). Contrastingly, Gomez et al. found negligible correlation with some NIRS parameters and acceptable agreement with others (Gomez et al., 2022). VOP is considered to represent tissue flow while NIRS interrogates only a small region of interest (∼1 cm^3^) which could explain inconsistencies between studies. Furthermore, the technical limitations in NIRS, such as variation in the contribution of Mb to the signal that would not impact VOP are likely to play a role in the discrepant findings.

Flow mediated dilatation (FMD) non-invasively assesses conduit vessel diameter up-stream of an arterial occlusion using ultrasound and, the extent to which the vessel can dilate post-ischemia is thought to largely depend on endothelial function. Downstream vasodilation is governed by multiple mechanisms (described above), all of which contribute to the NIRS signal. A positive correlation between reperfusion rate measured using NIRS and FMD has been shown in young, healthy men and in PAD patients (Cheatle et al., 1991; McLay et al., 2016a; Chung et al., 2018). Nevertheless, NIRS-measured PORH and FMD probably provide different but complementary information on the vascular response to ischemia.

## 6 Summary

We describe the application of NIRS for the assessment of PORH in skeletal muscle. We found no consensus on basic methodological approaches such as device sampling frequency, correction for adipose tissue, or analytical endpoints that should be calculated. Some areas for future research include: establishing the impact of variations in occlusion duration and comparing methods of accounting for different ischemic stimuli, the best method to account for ATT, the impact of different devices and establishing standards for minimum sampling rates. Addressing these methodological considerations should improve our understanding of PORH in skeletal muscle and facilitate interpretation of results across studies.
